# A Piezoresistive-Sensor Nonlinearity Correction on-Chip Method with Highly Robust Class-AB Driving Capability

**DOI:** 10.3390/s24196395

**Published:** 2024-10-02

**Authors:** Kai Jing, Yuhang Han, Shaoxiong Yuan, Rong Zhao, Jiabo Cao

**Affiliations:** 1 School of Automation and Information Engineering, Xi’an University of Technology, Xi’an 710048, China; yuhanghan2003@163.com (Y.H.); yuanshaoxiong2002@163.com (S.Y.); 15229001075@163.com (R.Z.); 18719725036@163.com (J.C.); 2 Xi’an Drawing Chips Corporation, Xi’an 710048, China

**Keywords:** piezoresistive sensor, nonlinear calibration, folded-cascode amplifier, Class-AB output op-amp

## Abstract

This paper presents a thorough robust Class-AB power amplifier design and its application in pressure-mode sensor-on-chip nonlinearity correction. Considering its use in piezoresistive sensing applications, a gain-boosting-aided folded cascode structure is utilized to increase the amplifier’s gain by a large amount as well as enhancing the power rejection ability, and a push–pull structure with miller compensation, a floating gate technique, and an adaptive output driving limiting structures are adopted to achieve high-efficiency current driving capability, high stability, and electronic environmental compatibility. This amplifier is applied in a real sensor nonlinearity correction on-chip system. With the help of a self-designed 7-bit + sign DAC and a self-designed two-stage operational amplifier, this system is compatible with nonlinear correction at different signal conditioning output values. It can also drive resistive sensors as small as 300 ohms and as high as tens of thousands of ohms. The designed two-stage operational amplifier utilizes the TSMC 0.18 um process, resulting in a final circuit power consumption of 0.183 mW. The amplifier exhibits a gain greater than 140 dB, a phase margin of 68°, and a unit gain bandwidth exceeding 199.76 kHz. The output voltage range spans from 0 to 4.6 V. The final simulation results indicate that the nonlinear correction system designed in this paper can correct piezoresistive sensors with a nonlinearity of up to ±2.5% under various PVT (Process–Voltage–Temperature) conditions. After calibration by this system, the maximum error in the output voltage is 4 mV, effectively reducing the nonlinearity to 4% of its original value in the worst-case scenario.

## 1. Introduction

Sensors can detect various physical quantities from the external environment (such as pressure, temperature, humidity, etc.) and convert them into usable electrical signals to facilitate information transmission, storage, and processing. Resistive sensors transform their corresponding variant output voltages to the following systems by utilizing external physical quantities to change resistance values. Common resistive sensors include strain gauge sensors, magnetoresistive sensors, temperature resistive sensors, piezoresistive sensors, and piezoelectric sensors. Among them, ferroelectric and piezoelectric sensors offer higher performance in specific applications due to their high sensitivity and superior adaptability to environmental changes [1,2,3,4,5]. Piezoresistive sensors hold a significant position in the sensor market due to their broad range of applications. They are not only used in consumer electronics, such as wearable devices, automotive safety detection systems, imaging, and medical diagnostic instruments [6,7,8,9], but also play an indispensable role in industrial fields, such as underground exploration and environmental monitoring.

As the primary component for collecting external physical information, the performance of sensor systems directly influences the overall system’s operational state [10]. However, the inherent physical nonlinearity of silicon wafers during sensor manufacturing, along with mismatched bridge arm resistances, result in a bow-shaped nonlinear conversion issue during the pressure-to-electrical signal conversion process [11]. This nonlinearity makes it difficult to precisely adjust the device’s operational state, which is especially critical in some applications. These applications require high linearity, where the linearity index can significantly impact the entire system’s functionality and data acquisition accuracy, such as in human muscle movement detection, continuous blood pressure monitoring devices, and deep-well pressure-sensitive sensing systems [12,13,14,15]. Therefore, the nonlinear calibration of piezoresistive sensors is crucial for ensuring their accuracy and reliability.

Nonlinear errors caused by the sensor’s design and structure are present from the time of manufacturing and are challenging to optimize. As a result, performing calibration experiments on the sensor and collecting interference factors for error compensation has become the mainstream approach for sensor correction [16]. In piezoresistive sensor correction systems, two main design facts must be addressed for industrial pressure transmitters to attain the desired pressure–voltage performance. One fact is the necessity for low-noise signal detection, during which the AFE (analog front-end) must ensure precise and low-noise data acquisition, allowing for the detection and noise-free sampling of minor deformations in the silicon core. The second fact is that the impedance of piezoelectric silicon products can vary greatly across different applications, ranging from 300 ohms to over ten thousand ohms. Maintaining stable signal detection and ensuring accurate readings across these varying impedance ranges presents a significant technical challenge.

The common compensation methods are generally categorized into software and hardware compensation [17]. Software compensation adjusts nonlinearity errors through algorithms, such as by establishing mathematical models and optimizing them to reduce the errors. On the other hand, hardware compensation directly addresses errors at the hardware level, eliminating the need for complex calculations and offering better real-time performance. Hardware compensation is further divided into board-level and on-chip compensation. Board-level compensation typically requires a larger footprint, consuming more PCB space, more power, and adding weight. Based on these considerations, this paper proposes an on-chip hardware compensation method. A balanced bridge is established by designing a Wheatstone bridge structure in the sensor’s measurement circuit to achieve nonlinear compensation for the piezoresistive sensor. To achieve precise, low-noise data acquisition and robust power-driving capability for various applications, a self-designed 7-bit + sign DAC and a self-designed two-stage operational amplifier are employed.

This paper is organized as follows. In Section 2, an experiment shows the fact that there is nonlinearity in the piezoresistive sensor. A nonlinear calibration model is proposed to solve this problem. Section 3 and Section 4 explain the main parts of the model, respectively, the DAC design and operational amplifier design. Section 5 gives some simulation test results. The conclusion is presented in Section 6.

## 2. Proposed Nonlinear Correction Structure of Piezoresistive Sensor

### 2.1. Silicon Voltage Detection Experiment

**Figure 1:**
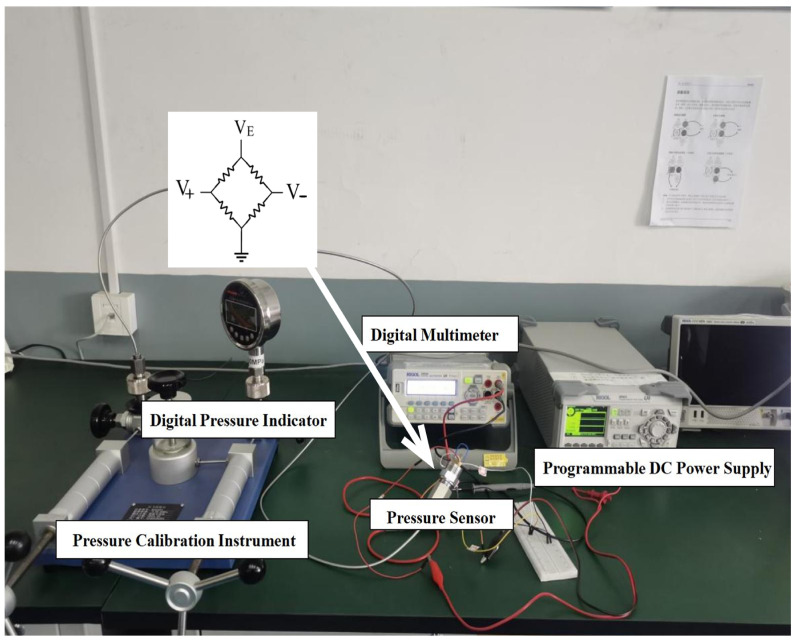
Silicon voltage detection experiment.

Due to the physical undesirable characteristics in thin film resistors and the resistance mismatch in the bridge–arm structure, a bow P-V (pressure-to-voltage) nonlinear transfer curve manifests in the process of pressure converting. Figure 1 shows a strain silicon voltage detection design (@room temperature). Pressure ranging from 0 to 40 M Pa is applied using the Pressure Calibration Instrument, and the real-time pressure values can be accurately read from a high-precision Digital Pressure Indicator. For a strain gauge silicon output voltage value that is not yet signal conditioned, any fluctuation in the power supply will directly transfer and overwhelm the uV to mV level output voltage’s value. To overcome this problem, a programmable DC power supply forming a noise-free DC value (1.756 V) is utilized to power the silicon core through the “+” supply node.

To enhance the applicability of the calibration method, data were collected for three distinct products (AS113, KC11, and SIN-P300) from three companies. These products share a common measurement range of 0–10 MPa and were all subjected to identical testing conditions.

**Figure 2:**
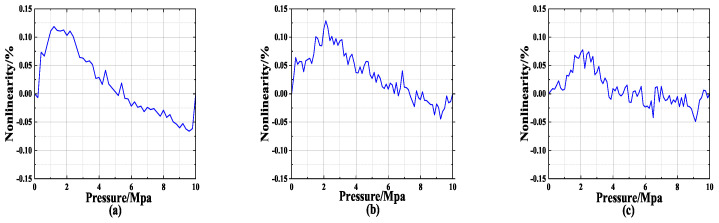
Silicon voltage detection design’s test results. (**a**) AS113 0–10 Mpa test results. (**b**) KC11 0–10 Mpa test results. (**c**) SIN-P300 0–10 Mpa test results.

As can be seen in Figure 2, a comparison of the nonlinearity across three different products within the range of 0–10 Mpa indicates that AS113 exhibits the worst performance. In industrial applications, extreme cases are often taken into account. If calibration can be achieved for the worst-case scenario, the overall performance nonlinearity becomes manageable. The overall performance nonlinearity becomes manageable if calibration can be achieved for the worst-case scenario. Therefore, AS113 will be the primary subject of subsequent research.

**Figure 3:**
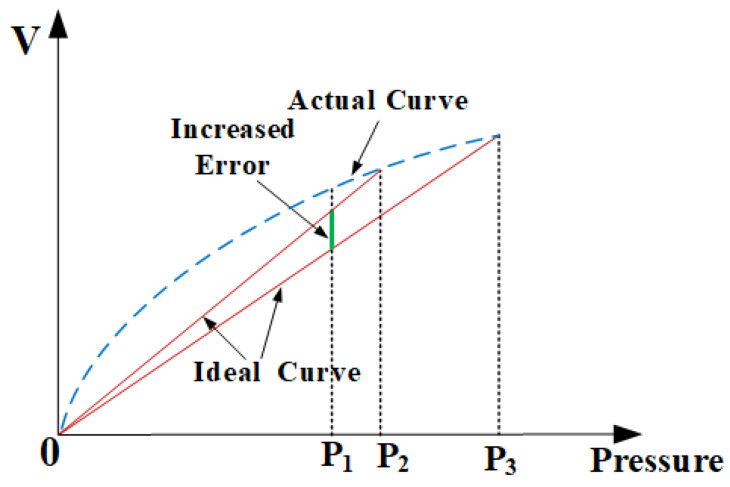
Nonlinearities across different measurement ranges.

**Figure 4:**
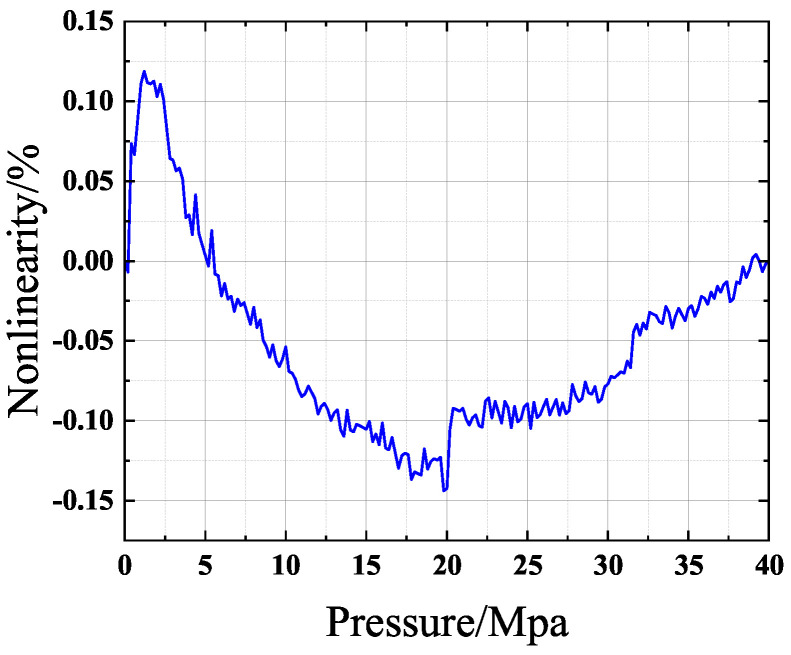
AS113 0-40Mpa test results.

Figure 3 illustrates the various nonlinearities observed across different measurement ranges. As depicted in Figure 3, $P_{1}$, $P_{2}$, and $P_{3}$ signify distinct pressure values. When the sensor’s measurement range is expanded from $P_{2}$ to $P_{3}$, the error between the actual curve and the ideal curve at $P_{1}$ will increase. Consequently, it is necessary to extend the sensor’s measurement range further to delve deeper into the sensor’s nonlinearity. Figure 4 presents the silicon output test results for the 40 MPa version of the AS113.

Figure 4 shows the silicon output test results for a certain product. As can be seen, the nonlinearity predominantly exhibits a downward-bowed trend across the entire measurement range, reaching its maximum at half of the full scale (20 Mpa). After curve fitting, the obtained expression between the voltage and external pressure reveals

(1) $$V=0.8523P+3.36\cdot 10^{-4}P^{2}-3.086\cdot 10^{-6}P^{3}+0.04127\;mV$$

In this equation, V represents the voltage value after fitting approximation, and P represents the external pressure value (P is dimensionless). Furthermore, the constant term (0.04127 mV) represents the result of zero-point drift (DC offset), and the first-order term corresponds to the desired output. In contrast, the second-order and third-order terms represent its nonlinear parts. As can be seen, the third-order term coefficient is approximately 1% of the second-order term coefficient, and the second-order terms occupy the main part. Consequently, coefficients of the third order and higher are typically neglected in subsequent investigations. Therefore, Equation (1) can be expressed as

(2) $$V=0.8523P+3.36\cdot 10^{-4}P^{2}+0.04127\;mV$$

In Equation (2), the constant and second-order terms are retained. During the process of converting pressure into voltage, the above equation can be expressed as

(3) $$V=V_{0}+V_{1}P+V_{2}p^{2}=V_{P}P_{E}$$

In Equation (3), $V_{P}$ represents the linear voltage during the process of converting pressure into voltage, while $P_{E}$ represents the nonlinear pressure output of the bridge with linear pressure input P, and the intrinsic nonlinear components of the sensor are embedded within $P_{E}$. Thus, the sensor’s nonlinearity issue is shifted to the process of pressure variation.

### 2.2. Proposed Nonlinear Correction Structure of Piezoresistive Sensor

Based on the above experiments, it can be concluded that in the absence of a feedback network within the system, errors would be amplified by the amplifier. Consequently, this paper proposes an on-chip solution to address this issue.

In this work, an effective model that can achieve nonlinear correction for sensors by using the designed amplifier previously is beyond its capability of providing robust driving functionality across varying sensor resistance values, so a nonlinear calibration mechanism utilizing minimal on-chip resources and components is implemented. This approach not only addresses the challenge of low-noise on-chip power driving but also significantly enhances the overall linear performance of the system only at a minimum chip area cost and power.

**Figure 5:**
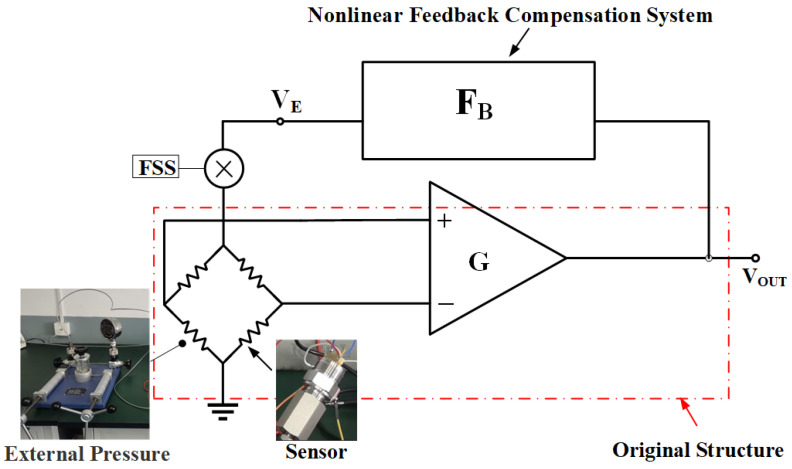
Schematic feedback compensation model.

In Figure 5, a schematic feedback compensation model is proposed, where $F_{B}$ represents a nonlinear feedback structure, which not only supplies the excitation voltage of the sensor $V_{E}$ but also achieves nonlinear feedback calibration and compensation. The “original structure” highlighted within the red box denotes the fundamental experimental structure presented in Figure 1. The resistance of the Wheatstone bridge varies with changes in the external pressure. The FSS represents the sensitivity of the electrical bridge in the process of converting pressure into an electrical signal. Based on the existing sensor and amplifier, after the pressure signal is converted into an electrical signal and amplified by the amplifier G, due to errors in the sensor, the output voltage $V_{OUT}$ undergoes nonlinear distortion, subsequently resulting in a corresponding alteration of its value. This model achieves nonlinear correction by acquiring the output voltage $V_{OUT}$, utilizing a feedback module $F_{B}$, and employing the resulting output $V_{E}$.

### 2.3. Model Establishment of $F_{B}$

As can be seen in Figure 5, $V_{OUT}$ can be expressed as

(4) $$V_{OUT}=V_{E}\cdot FSS\cdot P_{E}\cdot G\cdot V_{OUT}$$

In Equation (4), the intrinsic nonlinearity of the sensor is contained in $P_{E}$, and the value of the amplification factor of the amplifier G remains constant across all conditions. FSS is an essential conversion coefficient for transforming pressure into voltage output, which represents the full-scale bridge sensitivity of the sensor. Since the components of $P_{E}$ of the third order and above have been neglected in the preceding analysis, with the cancellation of $V_{OUT}$, Equation (4) can be represented as

(5) $$V_{E}=\frac{1}{FSS(AP+BP^{2})\cdot G}$$

**Figure 6:**
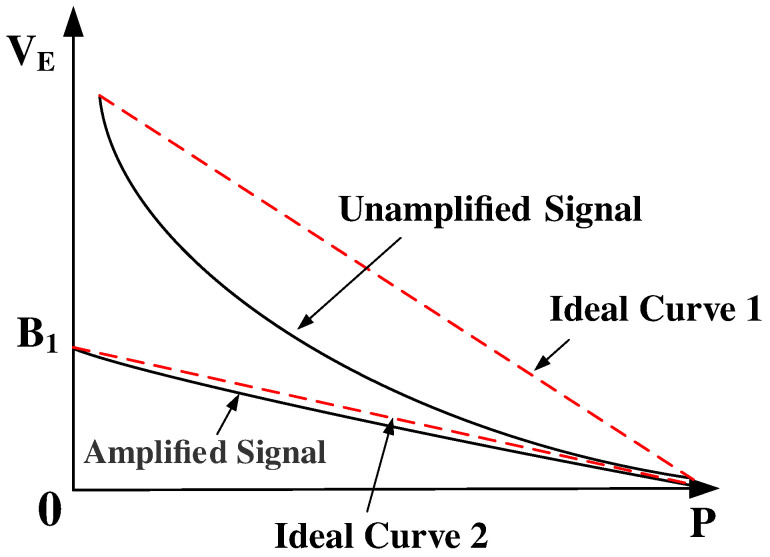
Unamplified and amplified first-order curve approximation.

For a specific type of $P_{E}$ (or specific nonlinearity), the internal structure of $F_{B}$ is adapted to the amplifier to eliminate the nonlinearity. Equation (5) is mathematically equivalent to an inverse proportional function curve scaled down by the value of G (normally ten to hundreds of times). As can be seen in Figure 6, the error between the unamplified signal curve and the first-order ideal curve 1 is significant, making it unfeasible to directly approximate it as a straight line. Fortunately, upon amplification by G, the amplified signal curve with the second-order term can be approximated as ideal curve 2. Since the input signal of $F_{B}$ structure is $V_{OUT}$, with the effect of the FSS, the pressure signal is converted into an electrical signal and amplified by G to output $V_{OUT}$, $V_{E}$ can be expressed as

(6) $$V_{E}=\frac{1}{FSS(AP+BP^{2})\cdot G}\approx A_{1}P+B_{1}$$

In Equation (6), $A_{1}$ represents the first-order coefficient, and $B_{1}$ represents the constant term. Furthermore, $A_{1}$ represents the slope of the ideal curve 2 in Figure 6, while $B_{1}$ denotes the y-intercept of the curve.

Under the designed circumstance (nonlinearity is approximately 0%), $V_{OUT}$ and P are related through a first-order linear relationship, which can be represented as

(7) $$V_{OUT}=C\cdot P$$

In Equation (7), C represents the first-order coefficient:

(8) $$V_{E}=\frac{A_{1}}{C}V_{OUT}+B_{1}=A_{2}V_{OUT}+B_{1}$$

**Figure 7:**
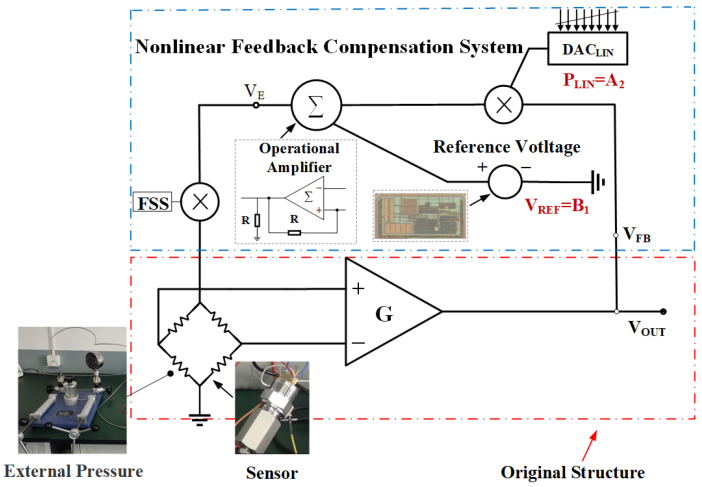
Detailed feedback implementation circuit.

In Equation (8), $A_{2}$ represents the first-order coefficient. Based on Equation (8), the internal structure of $F_{B}$ is presented as depicted in Figure 5. This is an innovative calibration model that adapts to the characteristics of different sensors by adjusting the value of $A_{2}$ and setting $B_{1}$ to a specific value. Based on Equation (8), the internal structure of $F_{B}$ is presented as depicted in Figure 7. As can be seen in Figure 7, $DAC_{LIN}$ can provide $A_{2}$ in Equation (6), which represents the linearization coefficient $P_{LIN}$. Since the value of $P_{LIN}$ necessitates adaptation to the varying nonlinearities of different sensors, it is tunable and of high precision. Furthermore, given that in actual scenarios, the curve may exhibit both downward-bowed and upward-bowed trends, the DAC requires a sign bit to accommodate these variations. The constant term $B_{1}$ in Equation (8) can be provided by the self-designed chip, which can offer a stable reference voltage $V_{REF}$ under different PVT (Process–Voltage–Temperature) conditions.

After multiplying $V_{OUT}$ by the linearization coefficient $P_{LIN}$ ($A_{2}$), it is summed by an operational amplifier with $V_{REF}$ ( $B_{1}$) provided by the self-designed chip to obtain the excitation voltage $V_{E}$ supplied to the sensor. $V_{E}$ can be represented as

(9) $$V_{E}=A_{2}V_{OUT}+B_{2}=P_{LIN}\cdot V_{OUT}+V_{REF}$$

In Equation (6), G is positioned in the denominator for $A_{1}$. Equation (7) illustrates that the conversion of pressure into the target voltage value is facilitated by the feedforward gain G, which functions as a coefficient in the numerator. Subsequently, Equation (8) indicates that $A_{2}$ is inversely proportional to 1/$G^{2}$, thus acting as its coefficient. Given the high amplification factor typical of amplifiers, the $A_{2}$ value is consequently quite small. Analysis and correction of the nonlinear characteristics across a broad spectrum of products reveal that $A_{2}$ must maintain an accuracy range between ±0.0125 and ±1, implying a requirement for over 80 discrete DAC levels. To ensure accuracy and balance chip area with power efficiency, a 7-bit DAC with a sign bit is used for $A_{2}$ value generation, offering 256 levels (128 positive and 128 negative) and allowing for precise correction. Additionally, since sensors exhibit both upward and downward bow-shaped nonlinearity, an extra sign bit is required. Furthermore, the operational amplifier G needs to have a powerful driving capability. In industrial applications, it is also important to consider factors such as area occupancy, cost, and power consumption. The detailed explanations of the designs of these two circuits components will be seen in the following sections.

## 3. Design of Current Steering DAC for Nonlinear Calibration

The traditional approach to designing the unit current source for current-steering DAC involves generating a reference voltage through a bandgap reference module, converting this voltage into a stable unit current source via a voltage-to-current (V-to-I) circuit, and then replicating this current source using current mirrors for internal circuit applications. However, in the context of nonlinear calibration DAC for piezoresistive sensors, the magnitude of the unit current source must dynamically adjust according to the varying output voltage of the sensor. To achieve this, the sensor’s output voltage is directly input into the V-to-I circuit after voltage division (i.e., $V_{FB}$ in Figure 7), thereby generating the necessary current source. Subsequently, by distributing this current source, the design of the adaptive unit current source tailored for the sensor’s nonlinear calibration requirements is realized.

**Figure 8:**
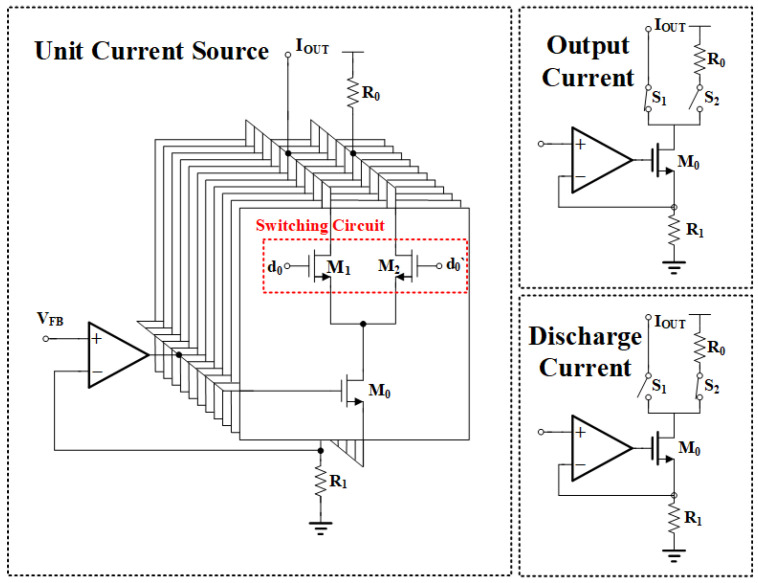
Unit current source.

Within a DAC circuit, a specific current source unit can be realized through current shunting, utilizing identically sized MOS transistors. As depicted in Figure 8, this current source unit employs MOS transistors $M_{1}$ and $M_{2}$, controlled by an 11-bit digital signal ($d_{0}$ and its complement $d_{0}^{\prime }$) generated by a segmented decoder. This control scheme facilitates two distinct operational modes: the output current mode and the discharge current mode. In the output current mode, when the digital signal activates $M_{1}$ and deactivates $M_{2}$, the current generated by the DAC current steering mechanism within this unit is directed solely towards the output terminal $I_{OUT}$. Conversely, in the discharge current mode, when the digital signal deactivates $M_{1}$ and activates $M_{2}$, the current generated by the same mechanism is fully diverted through resistor R0 for dissipation, thereby protecting the circuit from potential harm. The output current and discharge current structures are shown in the right part of Figure 8.

**Figure 9:**
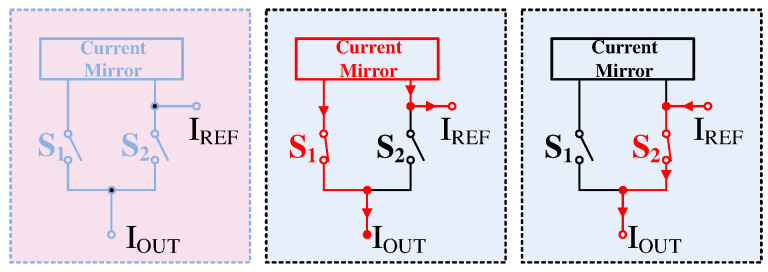
Ideal model for sign bits.

Since piezoresistive sensors exhibit both “upward-bowed” and “downward-bowed” nonlinearity issues, it is necessary to design a signal path with a polarity to meet the design requirements during the nonlinearity correction process. As shown in Equation (9), the linearization coefficient $P_{LIN}$ differentiates between positive and negative signs. Consequently, the DAC output current must support both sourcing and sinking currents. To achieve the effect of changing the current direction, the ideal circuit model is shown in Figure 9. In this model, switches $S_{1}$ and $S_{2}$ are always in opposite states. When $S_{1}$ is closed and $S_{2}$ is open, the current flows entirely into the current mirror, providing a source current to the output after duplication. Conversely, when $S_{2}$ is closed and $S_{1}$ is open, the current directly provides a sink current to the output.

In summary, the 7-bit + sign DAC (i.e., $DAC_{LIN}$ in Figure 7), composed of the unit current sources and the sign bit circuitry, successfully generates the linearization coefficient $P_{LIN}$ used in Equation (9).

## 4. Design of the Two-Stage Operational Amplifier with Class-AB Output Stage

### 4.1. Functional Structure Design of the Operational Amplifier

**Figure 10:**
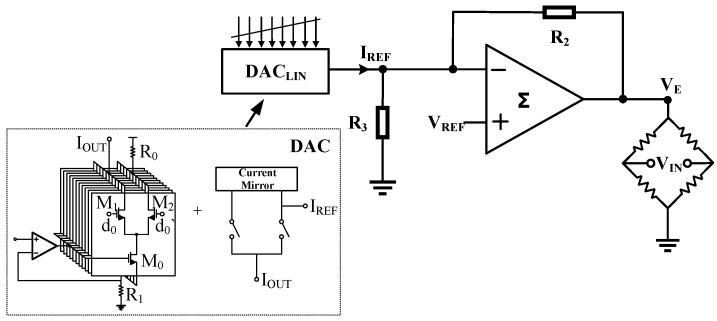
Functional diagram of the output-stage operational amplifier.

The primary function of the output-stage operational amplifier used for sensor nonlinearity calibration is to process the sensor’s output signal in conjunction with a reference voltage, thereby generating the excitation voltage $V_{E}$. The specific circuit principle is shown in Figure 10. In the figure, a DAC for nonlinearity calibration produces a reference current $P_{LIN}$. This current is converted into a corresponding voltage signal via resistor $R_{3}$. Subsequently, this voltage signal, along with an external reference voltage $V_{REF}$, are input into an operational amplifier for processing, resulting in the excitation voltage $V_{E}$. It is evident that the desired output voltage $V_{E}$ can be obtained by adjusting the linearization coefficient $P_{LIN}$ and the reference voltage $V_{REF}$ [18]. Based on the varying characteristics of different sensors, when a sensor detects an input pressure signal, it outputs a corresponding voltage signal. The nonlinear characteristics within this voltage signal are transmitted to the output stage via the amplifier. Meanwhile, the DAC adjusts its settings based on the feedback signal collected from the output (i.e., $V_{FB}$) to obtain an optimized linearization coefficient (i.e., $P_{LIN}$). This coefficient is then processed and used to modify the excitation voltage $V_{E}$ of the Wheatstone bridge, thereby compensating for the nonlinear characteristics. In summary, effective compensation for various sensors is achieved through the closed-loop nonlinear compensation network.

**Figure 11:**
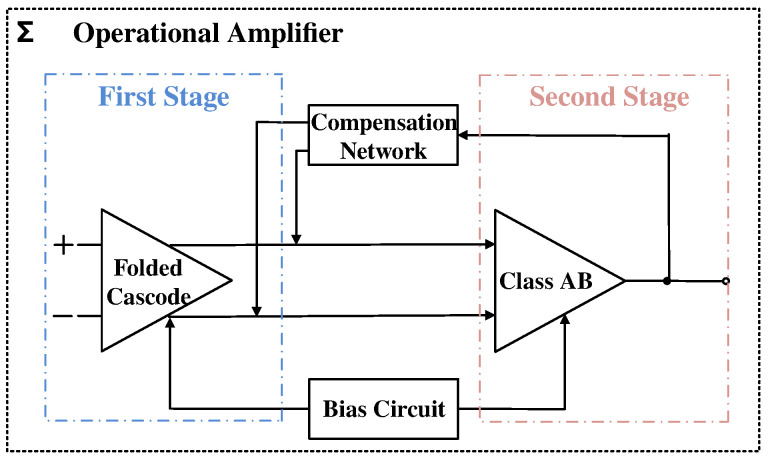
Two-stage operational amplifier structure.

This paper adopts a two-stage operational amplifier structure to balance gain and swing. The overall circuit block diagram is shown in Figure 11. The first stage consists of a folded cascode operational amplifier, and the second stage employs an improved Class-AB output structure. Additionally, the bias circuit provides appropriate bias voltages and currents to each component, and the compensation network cancels the zero–pole pairs through Miller compensation, thereby improving the stability of the circuit.

**Figure 12:**
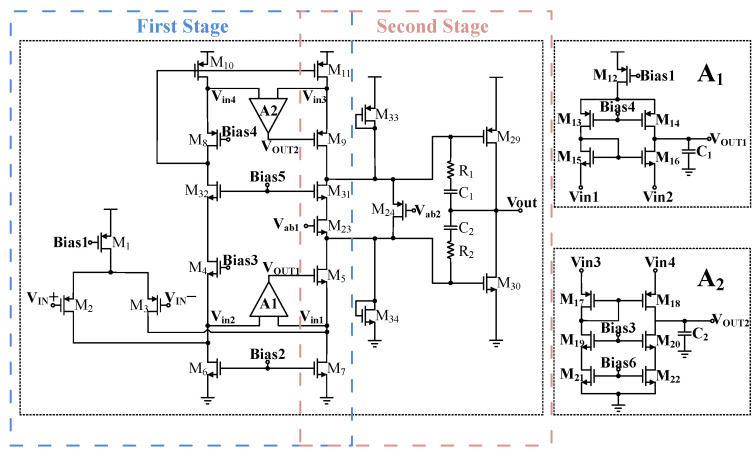
The specific circuit of the two-stage operational amplifier.

The specific circuit diagram of the two-stage operational amplifier is shown in Figure 12. The circuit implements a two-stage operational amplifier configuration, where the first stage uses a folded cascode amplifier primarily for amplifying voltage signals. The key performance parameters of this stage are gain and noise. Because the conventional folded cascode operational amplifier falls short of the design requirements, a trans-impedance amplifier is integrated to enhance gain. PMOS transistors are incorporated as the input differential pair to mitigate noise in the first stage. Given that the sensor linearization compensation system operates with a high-resistance load, a Class-AB output stage is selected for the second stage of the operational amplifier to balance efficiency with distortion-free performance. Additionally, an NMOS pair is introduced to the traditional floating gate voltage technique to stabilize the Class-AB output stage and further enhance the gain of the preceding stage. The detailed design methodology will be explained in subsequent sections.

**Figure 13:**
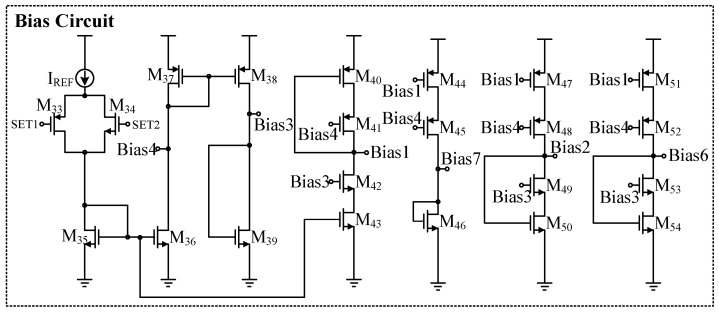
Bias circuit.

Figure 13 shows the overall bias circuit. This circuit utilizes the chip’s reference current and ensures proper operation of the bias circuit through the switch formed by $M_{33}$ and $M_{34}$. Self-biasing techniques are employed in the circuit to reduce system offset. The entire biasing circuit is placed in the same region of the chip to minimize the effects of process variations as much as possible. The bias circuit provides accurate bias voltages for the transistors in the two-stage operational amplifier.

### 4.2. Design of the First-Stage Operational Amplifier: Folded Cascode Operational Amplifier Structure

**Figure 14:**
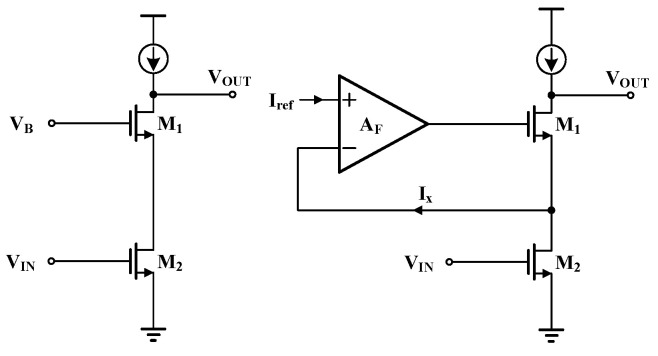
Two cascode amplifiers with different structures.

Since the first stage of the two-stage operational amplifier needs to amplify the voltage while ensuring low noise performance, gain is the primary consideration for this stage. The folded cascode amplifier, with its high output impedance and large output swing, is commonly used in the first-stage amplifier circuit. This paper introduces current-to-current negative feedback based on the traditional folded cascode amplifier to enhance the circuit gain by increasing the output impedance. The part of the common-source amplifier with current-to-current negative feedback to increase gain is shown in the right part of Figure 14. The amplifier $A_{F}$ in the figure serves the function of current-to-current negative feedback. Before introducing the negative feedback (as shown in the left part of Figure 14), the equivalent output impedance of the circuit is

(10) $$R_{out}\approx g_{m1}\cdot R_{o1}\cdot R_{o2}$$

In Equation (10), $g_{m1}$ is the equivalent transconductance of MOS transistor $M_{1}$, and $r_{o1}$ and $r_{o2}$ are the output equivalent resistances of MOS transistors $M_{1}$ and $M_{2}$, respectively.

After introducing current-to-current negative feedback, the equivalent transconductance of the original circuit remains unchanged, but the equivalent output impedance increases to [19]

(11) $$R_{out}\approx A_{add}\cdot g_{m1}\cdot R_{o1}\cdot R_{o2}$$

In Equation (11), $A_{add}$ is the gain of the negative feedback circuit $A_{F}$.

**Figure 15:**
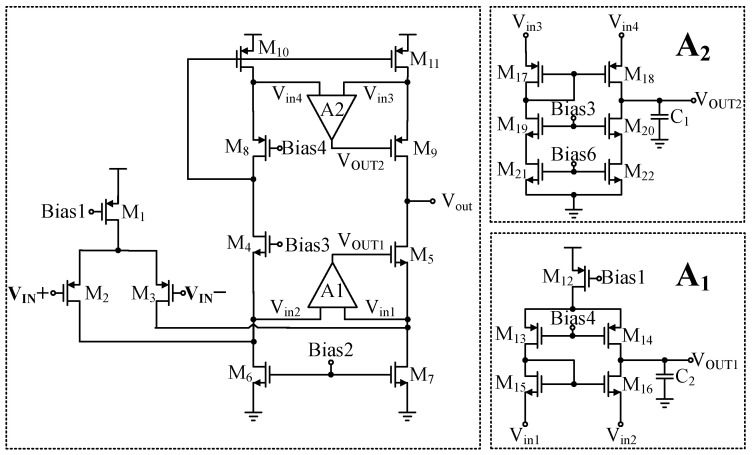
The first stage of the operational amplifier.

In summary, to achieve higher gain, this paper employs a cascode amplifier with current-to-current negative feedback. Additionally, considering the input stage’s requirements for large signal range and low noise, a folded cascode differential amplifier is employed. The overall folded cascode differential amplifier circuit with current-to-current negative feedback to improve gain is shown in Figure 15. After introducing two current-to-current negative feedbacks, the total gain of the designed operational amplifier is

(12) $$A_{v}\approx g_{m3}[\left(g_{m5}\cdot r_{o5}\left(r_{o5}\|r_{o7}\right)\left(A_{1add}+1\right)\right]\|\left[\left(g_{m9}\cdot r_{o9}\cdot r_{o11}\right)\left(A_{2add}+1\right)\right]$$

The conventional single-ended output folded cascode operational amplifier employs a low-voltage cascode current mirror structure. Owing to its asymmetric topology, there may be instances of current mismatch. To further increase the gain and enhance current matching performance, this work introduces two amplifiers, $A_{1}$ and $A_{2}$, which employ gain-boosting techniques. This strategy effectively improves current matching while satisfying the design requirement of gain enhancement without reducing the voltage headroom. Furthermore, PMOS transistors $M_{2}$ and $M_{3}$ are used as the input pair, which not only improves the second pole of the operational amplifier but also reduces noise.

### 4.3. Design of the Second Stage Operational Amplifier: Improved Class-AB Output Stage Circuit Structure

Since the sensor linearization calibration circuit needs to drive resistive loads, the varying load impedance can affect the circuit’s output characteristics. To ensure the stability of the sensor’s output stage, there are two key design metrics for the operational amplifier’s output stage: improving efficiency and minimizing distortion. The following equation gives the power efficiency of the amplifier:

(13) $$\eta =I_{L}/\left(I_{Q}+I_{L}\right)$$

In Equation (13), $I_{L}$ is the maximum current variation of the output stage, and $I_{Q}$ is the quiescent current of the output stage when unloaded.

To ensure the high power efficiency of the output stage, the output stage should be biased with a low quiescent current $I_{Q}$ while providing a large dynamic output current $I_{L}$. However, when the maximum output current variation $I_{L}$ is large, it may cause the MOS transistors to deviate from the saturation region, leading to an increase in nonlinear distortion. Therefore, there is a trade-off between efficiency and linearity in the output stage design.

Class-A output stages, although they have the advantages of high voltage gain, large output swing, and distortion-free performance, suffer from low output efficiency and are generally used for driving capacitive loads. In contrast, Class-B output stages offer large output swings, low quiescent currents, and high efficiency but suffer from significant crossover distortion [20]. Considering these factors, this design opts for a Class-AB output stage for the operational amplifier. The Class-AB configuration offers strong current driving capability and large output voltage swing while introducing minimal nonlinear distortion at relatively high efficiency. This characteristic can be further improved through compensation networks. Additionally, since the preceding folded cascode differential amplifier stage provides only a single output signal, this design uses floating gate voltage techniques to achieve the required level shifting to drive the Class-AB output stage.

**Figure 16:**
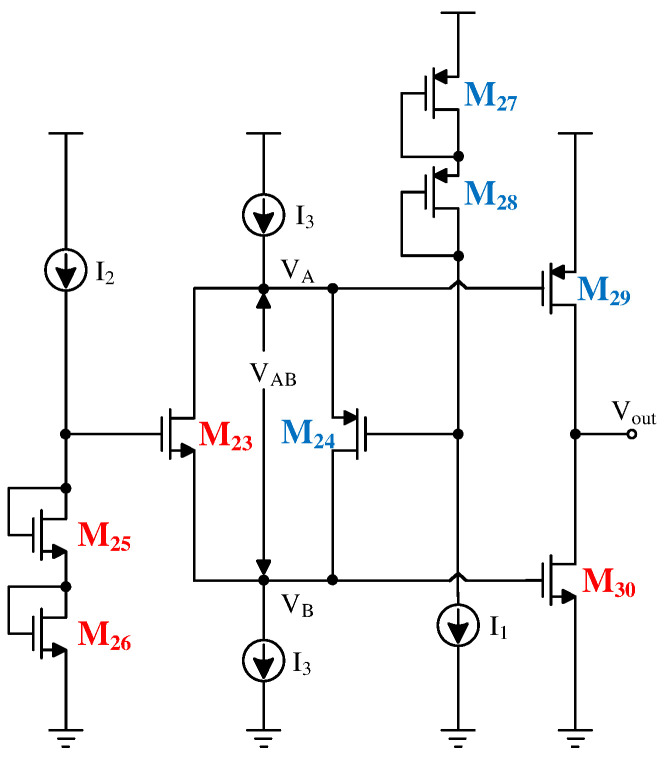
Traditional Class-AB output stage.

The traditional Class-AB output stage is shown in Figure 16. This structure consists of two translinear loops formed by $M_{23}$, $M_{25}$, $M_{26}$, $M_{30}$, and $M_{24}$, $M_{27}$, $M_{28}$, $M_{29}$, respectively. The static currents $I_{D29}$ and $I_{D30}$ of the output driver transistors $M_{29}$ and $M_{30}$ can be set by adjusting $I_{D24}$, ${\left(W/L\right)}_{24}$, $I_{D23}$, and ${\left(W/L\right)}_{23}$. The operating mode is as follows: when $V_{A}$ and $V_{A}$ rise simultaneously, the NMOS driver transistor $M_{30}$ conducts more, and the PMOS driver transistor $M_{29}$ tends to cut off due to the increase in gate voltage. According to Kirchhoff’s law, $M_{30}$ draws more charge from the load in this mode, thereby reducing the output voltage. As the gate voltages of $M_{23}$ and $M_{24}$ are fixed, |$V_{GS24}$| increases while |$V_{GS23}$| decreases, increasing the current through $M_{24}$ and decreasing the current through $M_{23}$, maintaining $\left[\sqrt{I_{D24}/\left({\left.W/L\right)}_{24}\right.}+\sqrt{I_{D29}/\left({\left.W/L\right)}_{29}\right.}\right]$ as a constant until the current through $M_{24}$ increases to $I_{3}$, at which point the drain source current through $M_{30}$ reaches its maximum value. When $V_{A}$ and $V_{B}$ drop simultaneously, the process is similar, with $M_{29}$ further conducting and $M_{30}$ tending to cut off, injecting current into the load from $M_{29}$, thereby achieving rail-to-rail output and providing a large output voltage swing.

**Figure 17:**
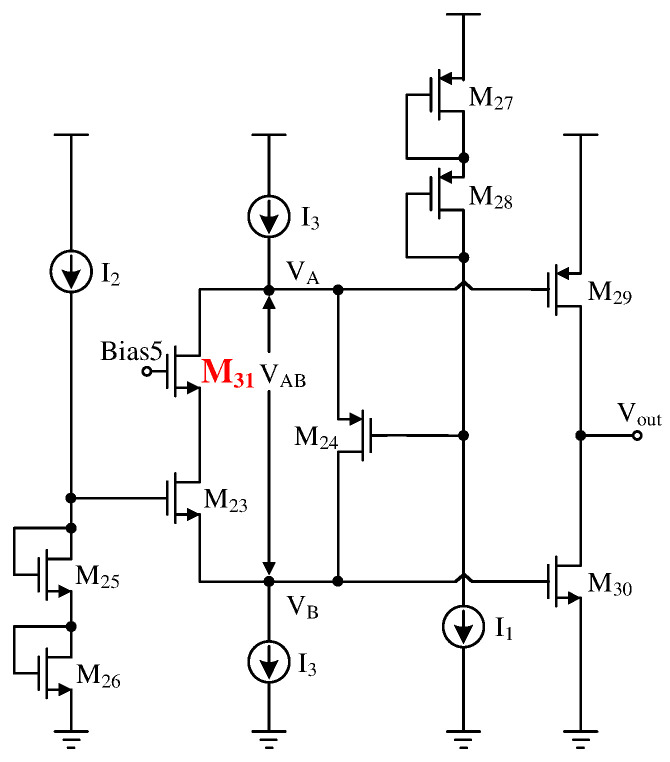
Improved Class-AB output stages.

Due to the different carrier mobilities of holes and electrons, PMOS transistors are more likely to enter the saturation region than NMOS transistors under the same current and drain-source voltage conditions. Therefore, in the process of injecting current with the traditional floating gate techniques, $M_{23}$ is prone to operate in the linear region, causing abnormal working conditions. To address this, the improved circuit topology shown in Figure 17 adds an NMOS transistor $M_{31}$ between the drain of $M_{23}$ and the source of $M_{24}$. Since the floating gate and the first-stage folded cascode share some common parts, $M_{32}$ is added to the first-stage folded cascode amplifier to ensure its normal operation as shown in Figure 12. Adding $M_{31}$ and $M_{32}$ reduces the output swing of the folded cascode amplifier and introduces additional poles. However, it improves the stability of the Class-AB output stage and the gain of the first stage. The first stage’s current-to-current negative feedback provides a sufficient voltage margin for the introduction of $M_{31}$ and $M_{32}$, and their inclusion does not alter the original working state of the floating gate.

### 4.4. Post-Simulation Results

**Figure 18:**
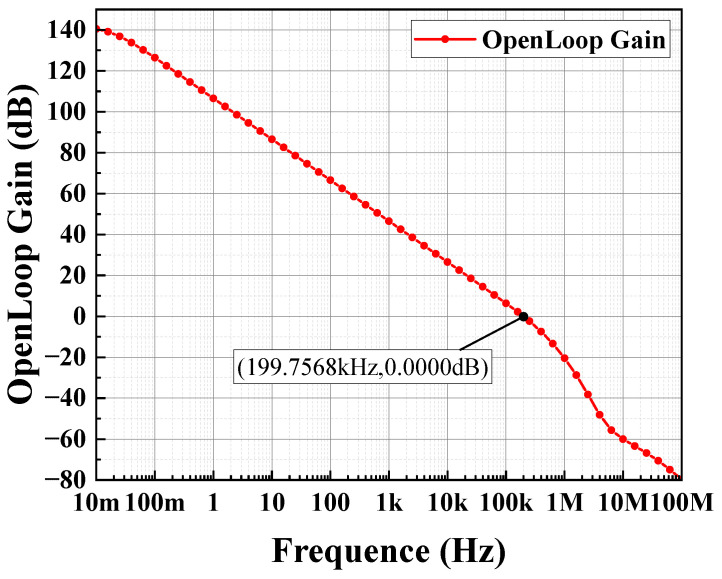
Output stage op-amp gain simulation results.

**Figure 19:**
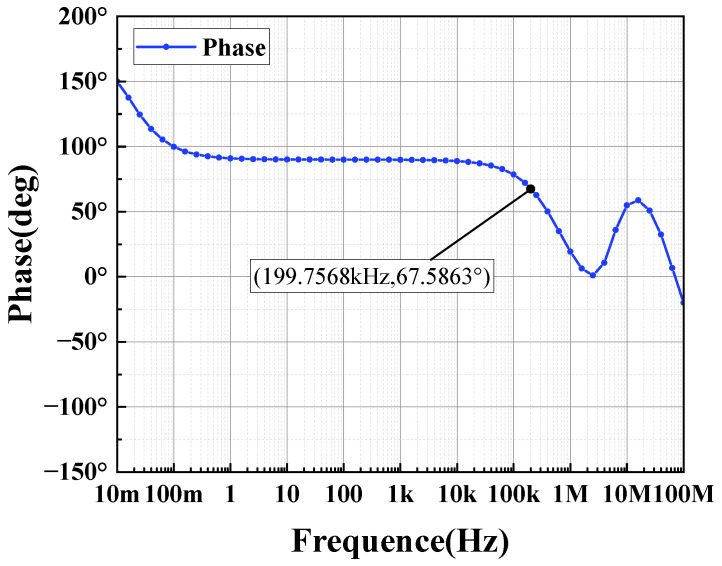
Output stage op-amp phase margin simulation results.

**Figure 20:**
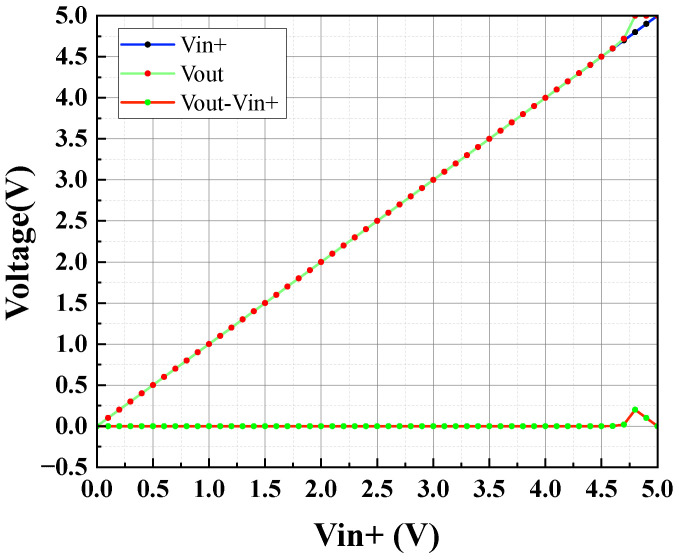
Output stage op-amp common-mode input range simulation results.

**Figure 21:**
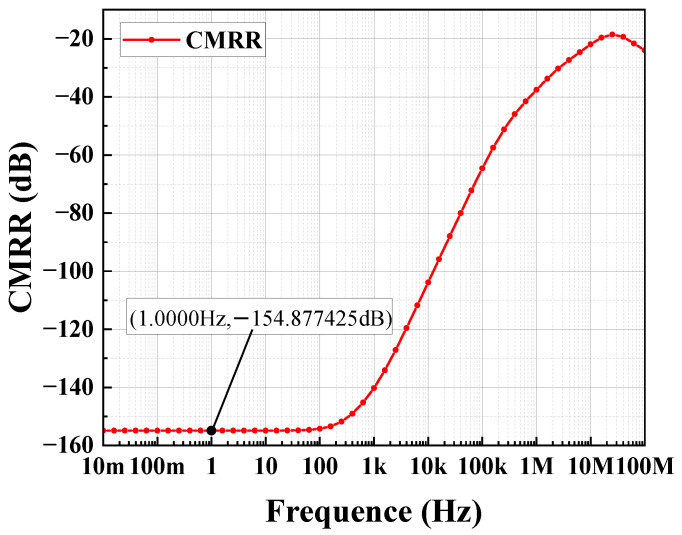
Output stage op-amp CMRR simulation results.

**Figure 22:**
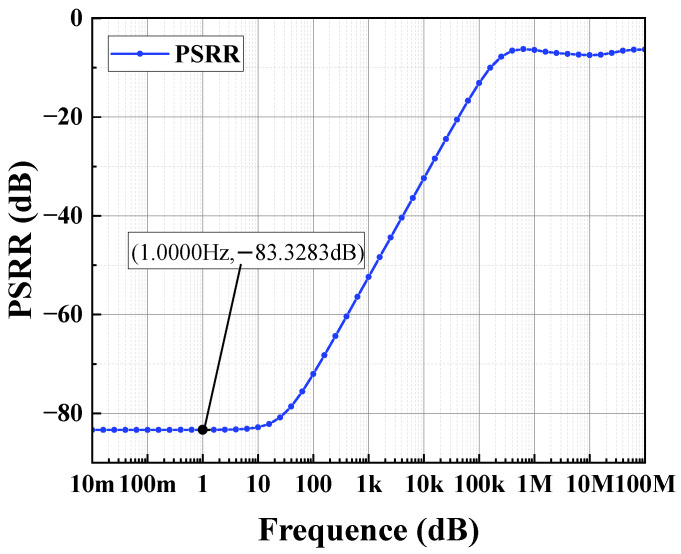
Output stage op-amp PSRR simulation results.

The circuit depicted in Figure 12 is simulated using Hspice with the TSNC 0.18 um CMOS process. The power supply voltage is set to 5 V, with a load capacitance of 100 pF. The amplifier’s amplitude and phase frequency responses are illustrated in Figure 18 and Figure 19. The operational amplifier achieves a gain exceeding 140 dB, a phase margin of 68°, and a unity gain bandwidth greater than 199.76 kHz. As shown in Figure 20, the common-mode input range spans from 0 to 4.6 V. Figure 21 and Figure 22 present the common-mode rejection ratio (CMRR) and power supply rejection ratio (PSRR), measured at 155 dB and 83 dB, respectively.

The overall simulation results of the operational amplifier are listed in Table 1. It can be observed that the operational amplifier designed in this work demonstrates excellent overall performance, including high open-loop gain, wide unity gain bandwidth, high common-mode rejection ratio (CMRR), high power supply rejection ratio (PSRR), and low power consumption.

**Figure 23:**
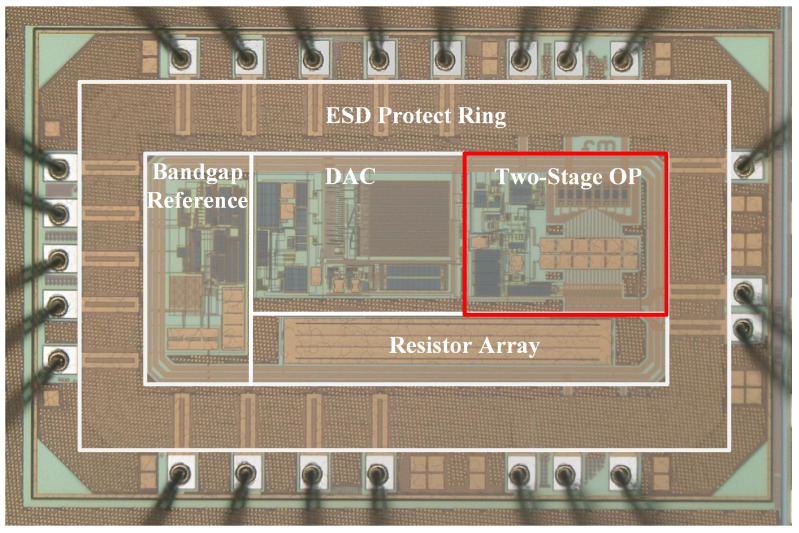
Image of the chip under a high-precision microscope.

Figure 23 shows an image of the chip after wafer fabrication, captured under a high-precision microscope. The layout structure of the operational amplifier designed in this work is shown in the red-boxed area of Figure 23. During the layout design process, a layout-versus-schematic (LVS) flow is used. A common-centroid matching method is employed for areas with high circuit matching requirements. The Length of Diffusion (LOD) technique is applied to stabilize the current. Considering the impact of process variations on the layout, dummy devices are incorporated into the design.

## 5. Test Results and Discussion

### 5.1. Measurements of the Current Steering DAC

**Figure 24:**
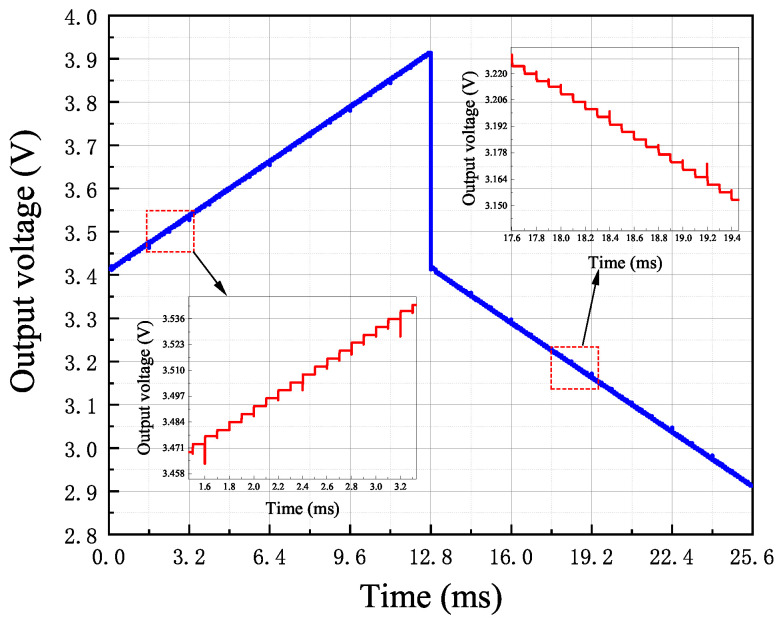
DAC conversion curve.

The simulation results of the DAC output signal as the digital signal increments sequentially are depicted in Figure 24. Due to the presence of the sign bit, the output curve of the current-steering DAC increases with the increment in the input signal’s bit positions in the first half, while it decreases with the increment in the digital signal’s bit positions in the latter half. As observed in the figure, there are 256 steps, indicating that the DAC resolution is 256. Furthermore, when the sensor’s nonlinear output voltage is 3 V, the DAC Least Significant Bit (LSB) is 3.92 mV. Additionally, larger glitches occur every eight steps, which arise during the transition from the binary code 1111 to 0000.

### 5.2. DAC Test Experiments

**Figure 25:**
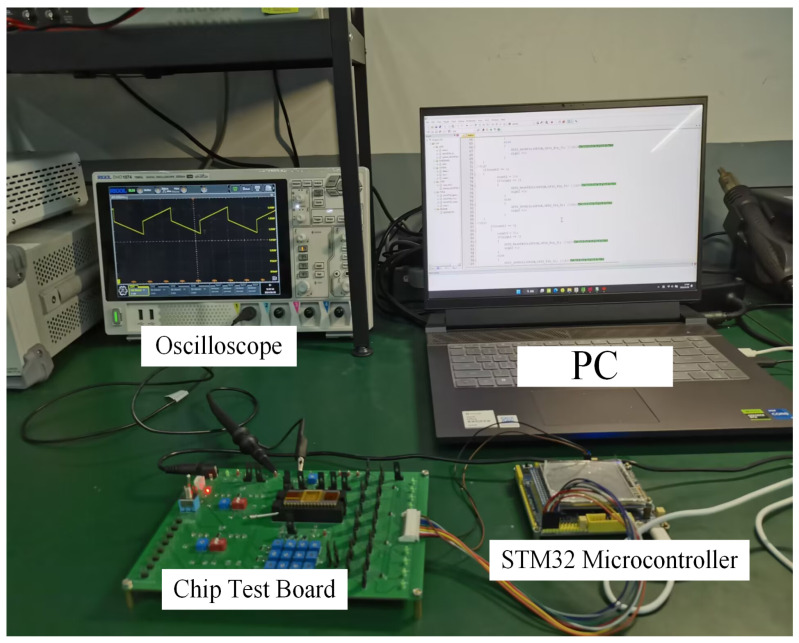
DACexperimental test environment.

**Figure 26:**
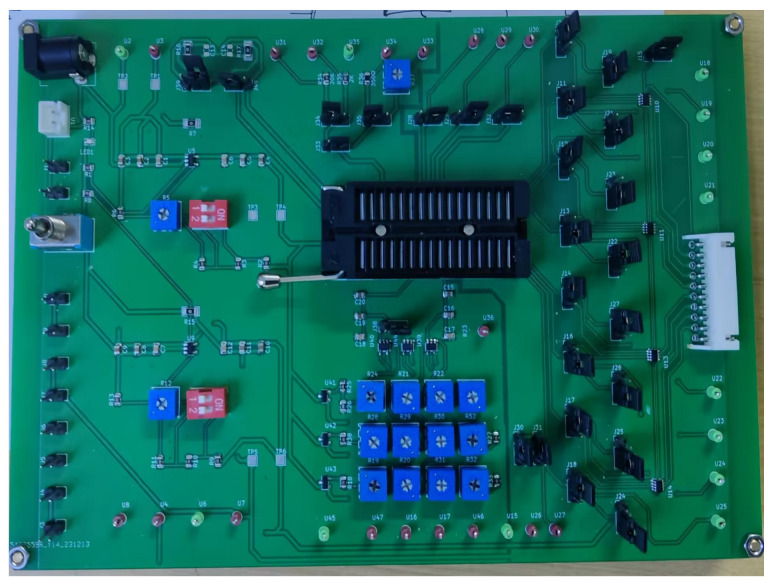
Chip test board.

The experimental setup for DAC testing can be seen in Figure 25. As can be seen in Figure 24, the STM32 microcontroller is connected to the PC and the chip test board. Code is developed on the PC to generate waveforms that simulate different cycles, which are then programmed into the STM32. Once connected to the digital port of the chip test board, it ultimately produces waveforms on the oscilloscope to verify the performance of the DAC. Adjustable resistors are employed to mimic the actual resistance values of a Wheatstone bridge, and resistance voltage division is used to simulate the output voltage of a sensor. The chip test board can be seen in Figure 26.

**Figure 27:**
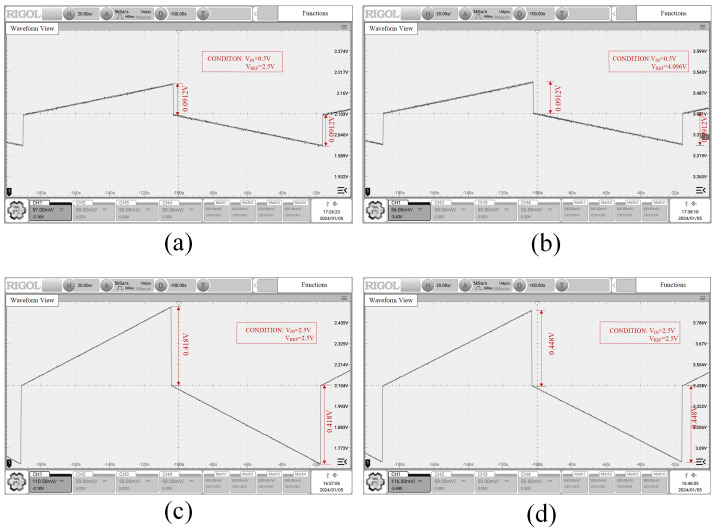
DAC simulation results (**a**) $V_{IN}$ = 0.5 V, $V_{REF}$ = 2.5 V (**b**) $V_{IN}$ = 0.5 V, $V_{REF}$ = 4.096 V (**c**) $V_{IN}$ = 2.5 V, $V_{REF}$ = 2.5 V (**d**) $V_{IN}$ = 2.5 V, $V_{REF}$ = 4.096 V.

Simulation results of the DAC under various input voltages and reference voltage conditions, observed through an oscilloscope, are presented in Figure 27. Upon testing, it is discovered that by adjusting the input voltage to ensure the compliance of the DAC output voltage and the reference voltage with the operational rules of the operational amplifier, the DAC functions properly when the output voltage of the operational amplifier falls within the range of 0.01 V to 4.99 V.

### 5.3. Simulation Test Results of the Nonlinear Calibration Function

**Figure 28:**
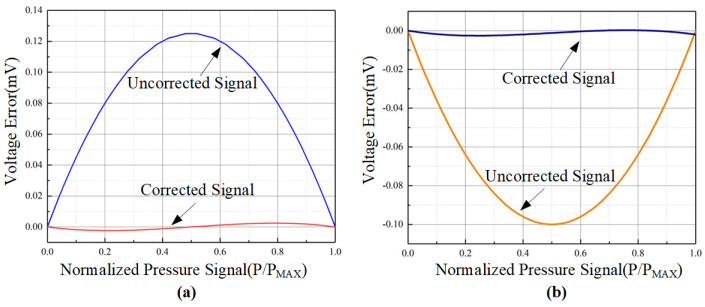
Correction results at a nonlinearity of 2.5%. (**a**) “Upward-bowed” nonlinear correction results. (**b**) “Downward-bowed” nonlinear correction results.

To simulate and verify the nonlinear correction functionality of a sensor, it is imperative to replicate the external environment. Additionally, simulations have been conducted for variables commonly encountered in actual circuits, encompassing power-on delay and variations in PVT, thereby ensuring a comprehensive portrayal of the actual operational environment. The nonlinear correction system designed in this paper is capable of correcting piezoresistive sensors with a nonlinearity of up to ±2.5%. Figure 28 shows the normalized version of these results, comparing the original and processed data, clearly indicating a significant improvement in nonlinearity.

**Figure 29:**
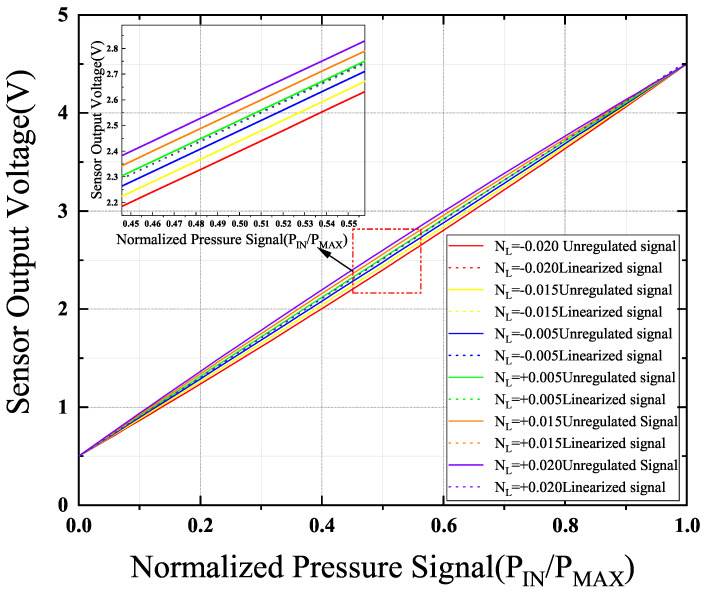
Correction results under different nonlinearities ($N_{L}$).

In Figure 29, the correction results of the calibration chip for different degrees of nonlinearity are shown. As can be seen in Figure 29, it illustrates the chip’s calibration of nonlinearity ($N_{L}$) ranging from −0.02 to +0.02 under various PVT conditions, demonstrating the chip’s calibration capability for the nonlinearity of different sensors. After calibration with this chip, the maximum error can be up to 4 mV, and the nonlinearity can be reduced to 4% of its original value in the worst case.

## 6. Conclusions

This paper addresses the issue of nonlinear distortion in sensors by proposing a correction model for piezoresistive sensor nonlinearity. This model is based on the underlying causes of the problem and the principles of calibration techniques. A specific circuit design method is proposed based on this model. The circuit mainly consists of three modules: a high-performance DAC, a high-drive capability output stage operational amplifier, and a reference circuit. The DAC section employs a 7-bit + sign DAC, and when the sensor’s nonlinear output voltage is 3 V, the DAC Least Significant Bit (LSB) is 3.92 mV. The output stage operational amplifier utilizes an improved folded cascode differential amplifier and a Class-AB push–pull output amplifier to achieve high gain and high output impedance. Leveraging the high efficiency, stability, and current gain characteristics of the Class-AB output stage, it can adaptively adjust the driving current capability. Additionally, on-chip Miller compensation technology is employed to adjust the frequency characteristics and ensure the stability of the operational amplifier. The designed output-stage operational amplifier is fabricated using the TSMC 0.18 um process under a power supply voltage of 5 V and a load capacitance of 100 pF. The final circuit power consumption is 0.183 mW. The operational amplifier features a gain exceeding 108 dB, a phase margin of 68°, a unity gain bandwidth greater than 1.9976 kHz, and an output voltage range from 0 to 4.6 V. Simulation results indicate that the nonlinearity correction system can calibrate piezoresistive sensors with nonlinearity up to ±2.5% under various PVT (Process–Voltage–Temperature) conditions. After calibration, the maximum output voltage error is 4 mV, effectively reducing nonlinearity to 4% of its original value in the worst-case scenario.

## Figures and Tables

**Table 1:** Simulation results of the amplifier parameters.

Parameter	Performance
Supply Voltage (V)	5
Phase Margin	68°
Input Common-Mode Range (V)	0–4.6
Power Consumption (mW)	0.183
Power Supply Rejection Ratio (dB)	83
Open-Loop Gain (dB)	140
Unity Gain Bandwidth (kHz)	199.76
Output Swing (V)	0–4.6
Common-Mode Rejection Ratio (dB)	155

## Data Availability

The data that support the findings of this study are available from the corresponding author, [K.J.], upon reasonable request.
